# Evaluating the Accuracy and Efficiency of Imaging Modalities in Guiding Ablation for Metastatic Spinal Column Tumors: A Systematic Review

**DOI:** 10.3390/cancers16233946

**Published:** 2024-11-25

**Authors:** Siran Aslan, Mohammad Walid Al-Smadi, Murtadha Qais Al-Khafaji, András Gati, Mustafa Qais Al-Khafaji, Réka Viola, Yousif Qais Al-Khafaji, Ákos Viola, Thaer Alnofal, Árpád Viola

**Affiliations:** 1 Department of Neurotraumatology, Semmelweis University, 1081 Budapest, Hungary; siran.aslan@phd.semmelweis.hu (S.A.); smadi996@hotmail.co.uk (M.W.A.-S.); 2 Doctoral School of Clinical Medicine, Semmelweis University, 1083 Budapest, Hungary; 3 Dr. Manninger Jenő National Traumatology Institute, 1081 Budapest, Hungary; murtadhaqais7@hotmail.com (M.Q.A.-K.); drgatiandras@gmail.com (A.G.); mustafaqais27@hotmail.com (M.Q.A.-K.); pallareka1004@gmail.com (R.V.); violaakos05@gmail.com (Á.V.); nofalthaer@yahoo.com (T.A.); 4 Department of Psychiatry, Peterfy Sandor Hospital, 1076 Budapest, Hungary; 5 Faculty of Medicine, University of Debrecen, 4032 Debrecen, Hungary; yousifqais7@hotmail.com; 6 Department of Cardiology, Ferenc Flór Hospital, 2143 Kistarcsa, Hungary

**Keywords:** spinal metastasis, thermal ablation, radiofrequency ablation, tumor control, microwave ablation, image guidance, cryoablation, cone-beam CT (CBCT), nerve injury, CBCT-MRI fusion

## Abstract

Background/Objectives: Spinal metastases are a frequent and serious complication in cancer patients, often causing severe pain, instability, and neurological deficits. Thermal ablation techniques such as radiofrequency ablation (RFA), microwave ablation (MWA), and cryoablation (CA) have emerged as minimally invasive treatments. These techniques rely on precise imaging guidance to effectively target lesions while minimizing complications. This systematic review aims to compare the efficacy of different imaging modalities—computed tomography (CT), magnetic resonance imaging (MRI), fluoroscopy, and mixed techniques—in guiding thermal ablation for spinal metastases, focusing on success rates and complications. Methods: A systematic literature search was conducted across PubMed, OVID, Google Scholar, and Web of Science databases, yielding 3733 studies. After screening, 51 studies met the eligibility criteria. Data on success rates, tumor recurrence, complications, and patient outcomes were extracted. Success was defined as no procedure-related mortality, tumor recurrence or expansion, or nerve injury. This systematic review followed PRISMA guidelines and was registered with PROSPERO (ID: CRD42024567174). Results: CT-guided thermal ablation demonstrated high success rates, especially with RFA (75% complete success). Although less frequently employed, MRI guidance showed lower complication rates and improved soft-tissue contrast. Fluoroscopy-guided procedures were effective but had a higher incidence of nerve injury and incomplete tumor control. Mixed imaging techniques, such as CBCT-MRI fusion, showed potential for reducing complications and improving targeting accuracy. Conclusions: CT remains the most reliable imaging modality for guiding thermal ablation in spinal metastases, while MRI provides enhanced safety in complex cases. Fluoroscopy, although effective for real-time guidance, presents limitations in soft-tissue contrast. Mixed imaging techniques like CBCT-MRI fusion offer promising solutions by combining the advantages of both CT and MRI, warranting further exploration in future studies.

## Simple Summary

Spinal metastases are a serious complication in cancer patients, often leading to pain and damage to the spine. One way to treat these metastases is by using special techniques that destroy the tumors with heat, a process known as thermal ablation. To do this safely and effectively, doctors use imaging tools like CT scans, MRI, or X-rays to guide the procedure. In this review, we compared these different imaging methods to determine which ones work best for guiding thermal ablation. We found that CT is commonly used and works well, but combining CT with MRI could improve safety by helping doctors see the tumor better and avoid nearby nerves. This research may help doctors choose the best imaging tools for treating spinal metastases, improving patient outcomes, and reducing risks.

## 1. Introduction

Spinal metastases commonly arise from the hematogenous or lymphatic spread of primary tumors, particularly in advanced stages of cancer [1]. Vertebral body metastases (VBMs) represent the most common type, accounting for 90% of spinal column lesions in cancer patients. [2]. Approximately 70% of VBMs affect the thoracic spine, 22% of the lumbosacral region, and 8% of the cervical vertebrae, with these lesions often disseminating hematogenously via Batson’s venous plexus [3]. Metastases frequently involve multiple segments of the spine [4].

Clinically, VBMs present with a wide range of symptoms, from localized back pain to metastatic spinal cord compression (MSCC), which can lead to severe neurological deficits due to vertebral body collapse or fracture [2]. Chronic pain, observed in about half of spinal metastasis cases, results from various factors such as direct tumor invasion, pathological fractures, pro-inflammatory cytokine release, and nerve root or spinal cord compression [5,6,7]. These symptoms often degrade patients’ quality of life and functional independence [4,8].

Open spinal biopsies, the gold standard for diagnosing spinal lesions, offer high diagnostic accuracy but come with increased risks of infection and morbidity due to their invasive nature [9,10,11,12]. In contrast, closed biopsy techniques, such as image-guided percutaneous biopsies, are less invasive and more cost-effective, utilizing modalities like fluoroscopy, ultrasound, CT, and MRI [13,14]. While advancements in closed biopsies aim to surpass open techniques, challenges remain in optimizing their accuracy and cost-effectiveness [12,14,15,16,17]. Despite these, the perfection and cost-effectiveness for improvement in closed biopsies to outperform the open biopsy techniques remain a big challenge [18]. Emerging technologies like cone-beam CT (CBCT)-MRI fusion have shown promise in enhancing precision, although larger studies are needed to confirm their clinical benefits [19].

Image-guided percutaneous thermal ablation (IPTA) is gaining traction as a minimally invasive treatment option for spinal metastases. By applying thermal energy, IPTA achieves tumor destruction with reduced morbidity compared to traditional approaches [20]. Techniques like ethanol ablation, cementoplasty, laser photocoagulation, and cryoablation have been utilized, each with its advantages and limitations [20,21].

Minimally invasive techniques have become essential in managing spinal vertebral metastases, including radiofrequency ablation (RFA), microwave ablation (MWA), cryoablation (CA), and spinal laser interstitial thermal therapy (SLITT). RFA and MWA effectively alleviate pain with minor complications reported, thus maintaining a generally safe profile [22,23]. Cryoablation shows promise in pain management. However, specific studies are scarce focusing on its application for spinal metastasizes [24]. On the other hand, SLITT demonstrates effectiveness in treating spinal metastases, particularly in cases of epidural spinal cord compression [25].

With precision and effectiveness in prohibiting the local growth of tumors, it is considered to be the promising treatment modality for spinal tumors. Traditional imaging modalities such as CT, fluoroscopy, and ultrasound were employed for accurate tumor targeting with minimal injury to surrounding critical structures [26,27] for over a couple of decades.

Traditionally, imaging modalities such as CT, fluoroscopy, and ultrasound have been employed to target tumors while accurately minimizing damage to surrounding tissues [28]. Recent advances, such as CBCT-MRI fusion, have improved visualization and targeting accuracy, with potential applications in diagnosis and tumor ablation [19]. These fusion techniques have already successfully treated tumors in other organs, such as the liver and kidneys [29,30,31].

This systematic review aims to evaluate the role of different imaging modalities—CT, MRI, fluoroscopy, and mixed techniques—in guiding thermal ablation techniques (RFA, MWA, CA, and others) in treating spinal metastases. Specifically, we aim to compare each imaging modality’s success rates, complication profiles, and long-term outcomes to provide clinical insights into the most effective imaging approaches for guiding these procedures.

## 2. Materials and Methods

This systematic review was conducted per the Preferred Reporting Items for Systematic Reviews and Meta-Analyses (PRISMA) guidelines to ensure comprehensive reporting and methodological rigor [32].

### 2.1. Research Aim and Search Strategy

**Figure 1:**
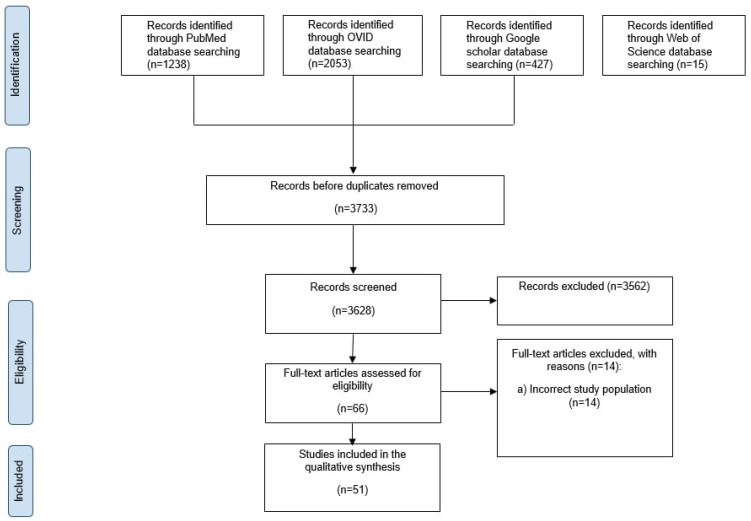
Schematic representation of study selection based on PRISMA.

The studies included were selected using a PRISMA-based framework (Figure 1). The review was registered in PROSPERO (ID: CRD42024567174). A predefined search protocol was followed, screening studies according to the following criteria:

- P (Population): patients aged 16 and over diagnosed with spinal column tumors;
- I (Intervention): thermal ablation;
- C (Comparison): conservative treatment;
- O (Outcome): the success rate of ablation, tumor recurrence, patient survival, complication rates, recovery time, procedural time, radiation exposure, and cost-effectiveness.

Databases searched included PubMed, OVID, Google Scholar, and Web of Science, covering studies from 2000 to 2024. A combination of MeSH terms, outlined in Table 1, was employed. Relevant studies were imported into Rayyan software (version 1.5.3) to identify and remove duplicates before proceeding with data review and study selection.

### 2.2. Selection Criteria

Inclusion and exclusion criteria were developed collaboratively among the research team to ensure a comprehensive and methodologically consistent study selection and data extraction. The inclusion criteria encompassed original research, observational studies, randomized controlled trials, and case series on spinal column tumors published between 2000 and 2024. Animal and cadaveric studies, meta-analyses, systematic and narrative reviews, case reports, editorials, conference proceedings, abstracts, and non-English studies with unavailable full texts were excluded.

### 2.3. Data Extraction and Management

Data were extracted using a standardized template adapted from the Cochrane Consumers and Communication Review Group. Extracted data included author names, databases, journals, publication dates, article types, DOI, titles, abstracts, methodology, and results. Two reviewers independently screened each record by title and abstract, with a third reviewer resolving any conflicts. Full texts were reviewed for all potentially relevant studies, and final decisions on inclusion were made independently.

### 2.4. Analysis and Synthesis of Data

A narrative synthesis was prepared based on the ablation success rate, tumor recurrence, patient survival, complication rates, postoperative recovery time, procedural time, radiation exposure, and cost-effectiveness. For the purposes of this review, the success rate was defined by the following criteria:

- No direct mortality related to the thermal ablation procedure.
- Absence of tumor recurrence or expansion at the treated site.
- No procedure-related nerve injury or other major complications.

Studies reporting outcomes that met all these criteria were considered to have a “complete success” rate.

### 2.5. Evaluation of the Studies

Full-text screening was performed using Microsoft Excel to record basic study details, including title, author, publication year, and eligibility criteria. Each study was scored based on its adherence to the inclusion criteria.

### 2.6. Reporting Guidelines

This review adhered to PRISMA guidelines for systematic reviews and meta-analyses and AMSTAR guidelines for assessing the methodological quality of systematic reviews.

### 2.7. Structure Overview

In this review, studies are categorized into four main groups based on the imaging modalities used to guide thermal ablation: fluoroscopy, CT, MRI, and mixed guidance. Each group is further divided into subsections that discuss the outcomes associated with different ablation methods within each imaging modality. This structure allows for a focused comparison of the efficacy and safety profiles of each imaging technique in guiding specific ablation procedures.

## 3. Results

Initially, our search strategy yielded 3733 studies collected from the three databases mentioned above (PubMed: 1238, OVID: 2053, Google Scholar: 427, Web of Science: 15). A total of 51 studies were removed after thorough abstract screening and duplicate removal (Figure 1).

### 3.1. Demographic and Clinical Data of All Patients/Study Selection and Overview

A total of 51 articles met the eligibility criteria and were reviewed. In total, 36 articles were retrospective [21,24,33,34,35,36,37,38,39,40,41,42,43,44,45,46,47,48,49,50,51,52,53,54,55,56,57,58,59,60,61,62,63,64,65,66], of which 2 articles were retrospective observational [67,68], 1 article was retrospective cohort [69], 5 articles were prospective [70,71,72,73,74], and 1 article for each of these studies was prospective observational [27], prospective cohort [75,76], prospective nonrandomized [77], and cohort study [76]. Three articles did not mention their study design [78,79,80]. Seven studies were multicenter [33,34,39,49,55,71,77], and the rest of the studies were carried out at a single institution.

A total of 1787 patients diagnosed with 2391 metastatic spinal lesions were analyzed. The mean age of all the patients was 60.45 years (age range: 16–89 years). Gender was reported in only 90.1% (*n* = 1611) of the patient population, with a similar ratio of males (*n* = 808) and females (*n* = 803), evaluated at a mean follow-up of 8.1 months. Thoracic spine was the most common location for spinal lesions occurring in 46.2% (*n* = 1041), followed by lumbar spine in 43.9% (*n* = 990), sacrum in 5.1% (*n* = 116), cervical spine in 3.7% (*n* = 83), coccyx in 0.6% (*n* = 14), ilium in 0.3% (*n* = 6), and periacetabulum in 0.2% (*n* = 4).

**Figure 2:**
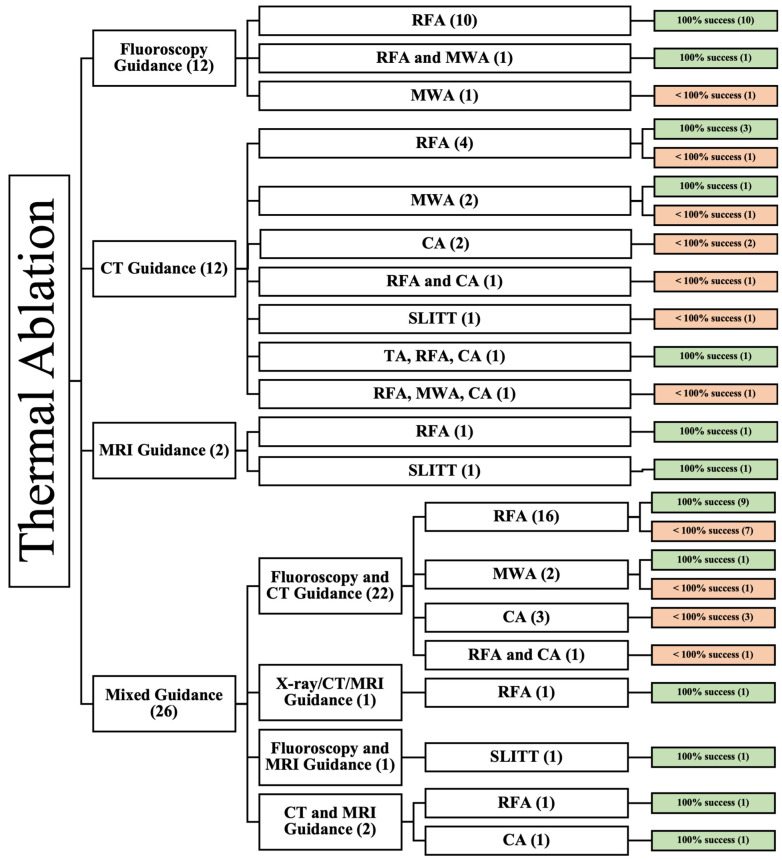
An overview of all patients undergoing image-guided TA. () the number of articles.

Twelve studies used fluoroscopy guidance [21,33,49,51,55,56,63,64,65,70,79,80], twelve used CT guidance [24,36,37,40,48,50,52,53,62,69,72,78], one used MRI guidance, and the rest of the twenty-six articles used a mix of guidance methods [27,34,35,38,39,41,42,43,44,45,46,47,54,58,59,60,61,66,67,68,71,73,74,75,76,77] (Figure 2).

### 3.2. Demographic and Clinical Data of Fluoroscopy-Guided Studies

**Figure 3:**
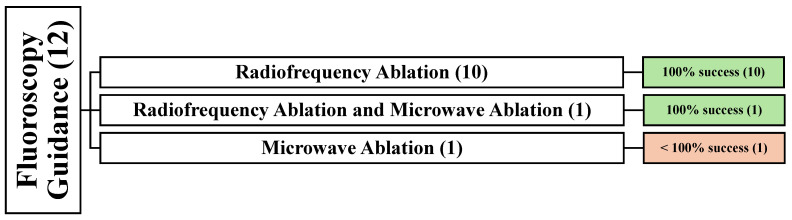
An overview of all patients undergoing fluoroscopy-guided techniques. () the number of articles.

Twelve articles reported using fluoroscopy guidance in 326 patients [21,33,49,51,55,56,63,64,65,70,79,80]. Among these, nine articles were conducted retrospectively [21,33,49,51,55,56,63,64,65], and one article was prospective [70]. However, the remaining two articles did not specify their study design [79,80]. Concerning the treatment modality, ten articles used RFA [21,49,51,55,56,63,64,65,70,80], one article used RFA and MWA [33], and another article used MWA [79] (Figure 3).

#### 3.2.1. Demographic and Clinical Data of Fluoroscopy-Guided RFA (10)

All ten articles [21,49,51,55,56,63,64,65,70,80] reported a 100% success rate. In total, 300 patients with 399 lesions were followed up for an average of 5.9 months. Eight of these articles [51,55,56,63,64,65,70,80] reported a mean age of 59.1 (range 18–89) for 158 male and 142 female patients included in these studies. Lung, breast, and kidney were the most common primary cancer sources (*n* = 108, 70, and 25, respectively). Spinal metastasis was reported in nine articles [21,49,51,55,56,63,64,70,80] and was most commonly located at the lumbar spine (*n* = 77), followed by thoracic (*n* = 69), sacral (*n* = 12), ilium (*n* = 3), and cervical spine (*n* = 2).

#### 3.2.2. Demographic and Clinical Data of Fluoroscopy-Guided RFA and MWA (1)

One article reported a 100% success rate using fluoroscopy-guided RFA and MWA [33]. This article involved 14 patients with 26 lesions that were followed up for a mean period of 6.7 months. The mean age of these patients was 67 years, including six male and eight female patients. Multiple myeloma and lung were the most common primary cancer sources (*n* = 5 and 4, respectively). Spinal lesions were found in the thoracic (*n* = 16) and lumbar (*n* = 10) spinal levels.

#### 3.2.3. Demographic and Clinical Data of Fluoroscopy-Guided MWA (1)

The last article [79], involving 12 patients with 12 lesions, revealed recurrence in 2 patients, resulting in an incomplete success of fluoroscopy-guided MWA due to nerve injury and recurrence. A mean age of 52.7 years (41–68 years) was reported in 12 females who were followed up for an average of 10.2 months. Breast cancer was the sole primary cancer etiology. All spinal lesions were located in the thoracic spine (Figure 3).

### 3.3. Demographic and Clinical Data of CT-Guided Studies (12)

**Figure 4:**
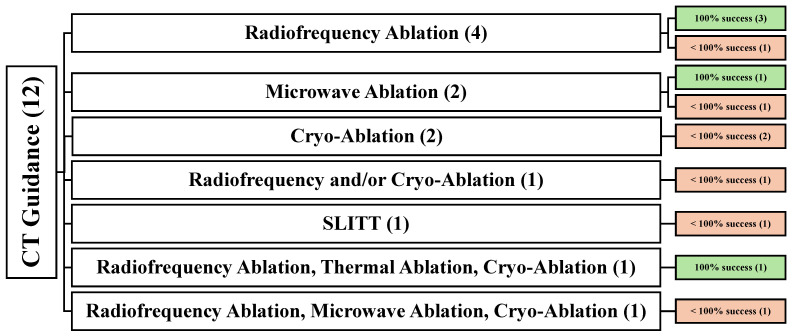
An overview of all patients undergoing CT-guided techniques. () the number of articles.

Twelve articles [24,36,37,40,48,50,52,53,62,69,72,78] reported using CT-guidance in all 424 patients who were involved. Among these articles, nine were retrospective [24,36,37,40,48,50,52,53,62], one was prospective [72], and one was retrospective cohort [69]. One article did not specify its study design [78]. In regard to the treatment modalities used, four articles used RFA [50,53,69,72], two articles used MWA [40,62], two articles used CA [24,52], one article used both RFA and CA [36], one article used SLITT [37], and two articles [48,78] used multiple treatment methods (TA, RFA, and CA [48], and RFA, MWA, and CA [78]) (Figure 4).

#### 3.3.1. Demographic and Clinical Data of CT-Guided RFA (4)

Three articles reported the complete success of CT-guided RFA in 43 patients with 53 lesions with a mean follow-up of 12.6 months (range: 3.6–36 months) [50,69,72]. A mean age of 61.5 years (37–84 years) was reported in 11 males and 32 females. Breast cancer was the most common primary cancer (*n* = 24), followed by colon and lung cancer (*n* = 5). The lesions of the spine were in the thoracic (*n* = 30), lumbar (*n* = 23), and sacral (*n* = 16) levels.

The last article [53], involving 26 patients with 28 lesions, revealed a local failure in 9 patients. Lung, breast, and esophageal cancer were the most common primary cancer etiology (*n* = 8, 8, and 3 patients, respectively). These patients were followed up for an average of 8.2 months. Mean age, age range, gender, and location were not reported in this article.

#### 3.3.2. Demographic and Clinical Data of CT-Guided MWA (2)

One retrospective study reported a 100% success rate of CT-guided MWA for 82 patients with 115 lesions [62]. The mean age was 63 years (range: 18–88 years), including 49 males and 33 females who were followed up for an average of 6 months. Lung cancer was the most common primary cancer etiology (*n* = 29), followed by breast cancer (*n* = 11), esophageal, and stomach cancer (*n* = 7 for both). Spinal lesions were located at thoracic and lumbar spinal levels (*n* = 48 and 67, respectively).

In contrast, the other retrospective study [40] reported neural complications, skin burns, and death due to heart attack, diffuse liver metastasis, and progression of upper thoracic spinal metastasis, resulting in an incomplete success of CT-guided MWA. This study involved 91 patients with 140 lesions, having a mean age of 62 years (range: 36–78 years), and it included 50 males and 41 females who were followed up for an average of 6 months. The most common primary cancer source was lung cancer (*n* = 36), followed by breast cancer (*n* = 13) and esophageal cancer (*n* = 11). Similarly, the spinal lesions were located at the thoracic and lumbar spinal levels (*n* = 71 and 69, respectively).

#### 3.3.3. Demographic and Clinical Data of CT-Guided CA (2)

Two retrospective studies reported the incomplete success of CT-guided CA for 25 patients with 44 lesions [24,52]. Postoperative radiculopathy of the lower extremities was a complication in both studies, with two patients each having this complication. The mean age was 53 years (range: 20–81 years), and it included 10 males and 15 females who were followed up for an average of 5.2 months (range: 3–13 months). Breast cancer was the most common primary cancer etiology (*n* = 4), followed by lung (*n* = 4) and colon cancer (*n* = 3). Spinal lesions were located at the cervical, thoracic, lumbar, sacral spinal, and coccygeal levels (*n* = 1, 10, 17, 12, and 2, respectively).

#### 3.3.4. Demographic and Clinical Data of CT-Guided RFA and CA (1)

Another study reported the complete success of CT-guided RFA and CA for 28 patients with 41 lesions [36]. The mean age was 60 years (range: 28–82 years), and it included 11 males and 17 females who were followed up for an average of 2.7 months (range: 1–6.6 months). Thyroid cancer was the sole primary cancer etiology (*n* = 28). Spinal lesions were located at the cervical (*n* = 5), thoracic (*n* = 17), lumbar (*n* = 16), and sacral (*n* = 3) spinal levels.

#### 3.3.5. Demographic and Clinical Data of CT-Guided SLITT (1)

Another study [37] reported local control failure and neurological complications, resulting in a lower success rate of CT-guided SLITT. A total of 110 patients with 120 lesions were involved in this study and followed up for an average of 7 months. Among these patients, 66 were males and 44 were females, and all had a mean age of 61.5 years. The most common primary cancers were renal, lung, and thyroid cancer (47, 13, and 10 patients, respectively). The location of the spinal lesions was reported in this article as well (C = 5, T = 107, L = 8).

#### 3.3.6. Demographic and Clinical Data of CT-Guided TA, RFA, and CA

One retrospective article reported the complete success of multiple treatment modalities (TA, RFA, and CA) guided by CBCT and angio-CT guidance [48]. In total, eight female patients with a mean age of 60 years (range: 42–75 years) were involved, and all their lesions were at thoracic level. They were all followed up for an average of 20 months (1–36 months). The primary cancer etiologies were breast (*n* = 6) and thyroid cancer (*n* = 2).

#### 3.3.7. Demographic and Clinical Data of CT-Guided RFA, MWA, and CA

Lastly, one study [78] reported vertebral fracture and an enlargement of the lesion’s volume, resulting in the incomplete success of multiple treatment modalities (MWA, RFA, and CA) guided by CT. In this study, a total of 11 patients with 12 lesions were involved and had lesions at the thoracic, lumbar, and sacral spinal levels (*n* = 6 each). The mean age of these patients was 62.9 years (49–76 years). They were all followed up for an average of 18 months (6–48 months). The primary cancer etiologies were breast (*n* = 2), lung (*n* = 2), and renal cancer (*n* = 2).

### 3.4. Demographic and Clinical Data of an MRI-Guided Study (1)

**Figure 5:**
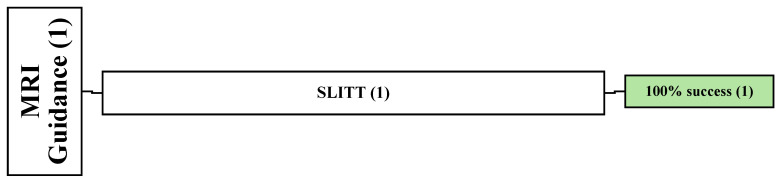
An overview of all patients undergoing MRI-guided technique. () the number of articles.

One retrospective article reported the complete success of MRI-guided SLITT in 11 patients with 11 lesions [57]. Among these patients, nine males and two females were involved, with a mean age of 56 years (range: 33–78 years), and were followed up for an average of 1.8 months. The most common primary cancers were renal cancer (*n* = 6) and pheochromocytoma (*n* = 2). The location of the spinal lesions was reported in this article (C = 1, T = 7, L = 3) (Figure 5).

### 3.5. Demographic and Clinical Data of Mixed Guidance Studies (26)

**Figure 6:**
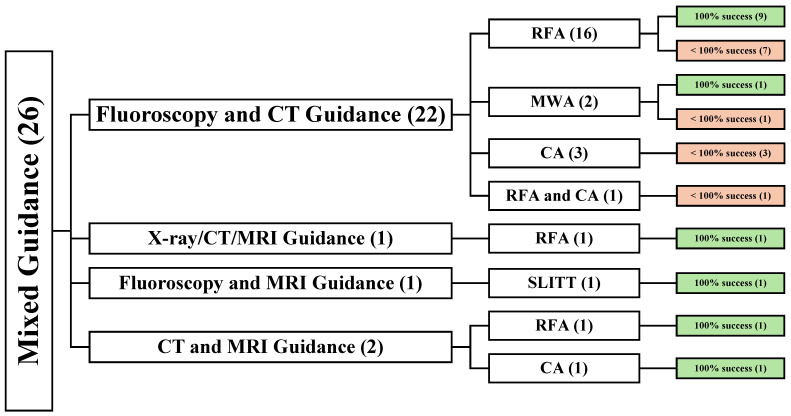
An overview of all patients undergoing mixed image-guided techniques. () the number of articles.

Twenty-six articles reported using different guidance techniques in 1026 patients with 1382 lesions [27,34,35,38,39,41,42,43,44,45,46,47,54,58,59,60,61,66,67,68,71,73,74,75,76,77]. Among these, seventeen studies were retrospective [34,35,38,39,41,42,43,44,45,46,47,54,58,59,60,61,66], three were prospective [71,73,74], two were prospective cohort [75,76], two were retrospective observational [67,68], one was prospective observational [27], and one was prospective nonrandomized [77]. Concerning the treatment modalities, twenty-two studies used fluoroscopy and CT guidance [27,34,35,38,39,41,42,43,44,45,46,47,54,59,60,61,67,68,71,73,75,77], two used CT and MRI guidance [74,76], one used X-ray, CT, and MRI guidance [66], and one used fluoroscopy and MRI guidance [58] (Figure 6).

#### 3.5.1. Demographic and Clinical Data of Fluoroscopy and CT-Guided Studies (22)

**Figure 7:**
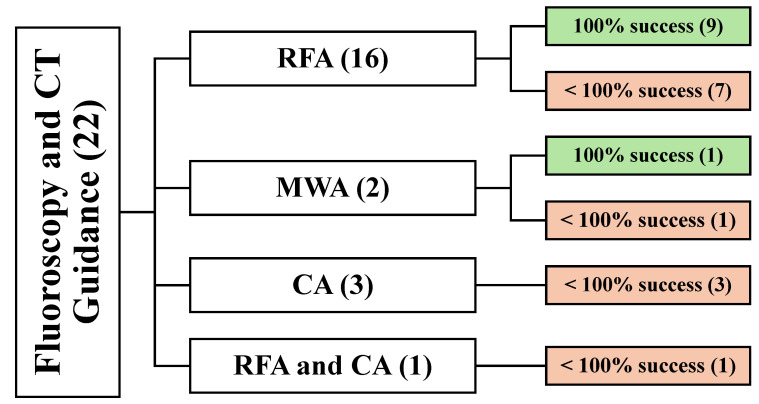
An overview of all patients undergoing fluoroscopy and CT-guided techniques. () the number of articles.

Twenty-two articles reported using fluoroscopy and CT-guided techniques. A total of 969 patients were involved, resulting in a total of 1310 lesions. The treatment modality was RFA in 16 articles [27,34,38,41,42,45,47,54,59,60,61,67,71,73,75,77], MWA in 2 articles [46,68], CA in 3 articles [35,39,43], and both RFA and CA in 1 article [44] (Figure 7).

##### Demographic and Clinical Data of Fluoroscopy and CT-Guided RFA (9)

Nine articles reported a 100% success rate for fluoroscopy and CT-guided RFA [27,34,38,41,47,60,61,75,77]. In total, there were 355 patients with 407 lesions. Seven of these articles [27,38,41,47,60,75,77] reported a mean age of 63.6 (range 30–89) for 107 male and 101 female patients who were included in these studies. Lung, breast, and kidney were the most common primary cancer sources (*n* = 76, 60, and 36, respectively). Seven articles [27,38,47,60,61,75,77] reported on the location of the spinal lesions (C = 3, T = 108, L = 130, S = 11, ilium = 4, and periacetabulum = 4). Six articles [27,34,38,61,75,77] reported a mean follow-up of 4.9 months.

In contrast, the seven remaining articles [42,45,54,59,67,71,73], which included 354 patients with 544 lesions, reported death [54], failure of local tumor control [59], local tumor progression [42], neural injury [45,71,73], residual metastasis, and recurrence [67], resulting in lower success rates for fluoroscopy and CT-guided RFA. The mean age for all patients was 61.3 (range: 21–87). A total of 170 male and 189 female patients were involved, with a mean follow-up of 5.2 months. The most common primary cancers were lung, breast, and genitourinary (*n* = 76, 68, 32, respectively). The location of the spinal lesions was given (C = 2, T = 206, L = 256, S = 30, and ilium = 3).

##### Demographic and Clinical Data of Fluoroscopy and CT-Guided MWA (2)

One study reported a 100% success rate for all 11 patients with 16 lesions who underwent fluoroscopy and CT-guided MWA [68]. The mean age was 65 years (range: 40–84 years), and it included seven males and four females who were followed up for 3 to 12 months. Lung cancer was the most common primary cancer etiology (*n* = 7), followed by breast cancer (*n* = 3) and thymoma (*n* = 1). Spinal lesions were located at the thoracic (*n* = 6) and lumbar (*n* = 5) spinal levels.

Another study [46] reported neural injury and skin burn, resulting in an incomplete success rate. This article involved 69 patients with 102 lesions who were followed up for 4.6 to 5.5 months. The mean age of these patients was 56 years, and the ratio of males to females was slightly higher (36 and 33 patients, respectively). The most common primary tumor etiologies were breast, lung, and multiple myeloma (*n* = 27, 12, and 10, respectively). The metastasis was in the cervical (*n* = 2), thoracic (*n* = 50), lumbar (*n* = 34), and sacral (*n* = 16) spinal vertebras.

##### Demographic and Clinical Data of Fluoroscopy and CT-Guided CA (3)

Three retrospective articles reported the failure of neural injuries [35,43], local tumor control [39], and recurrence [39], resulting in the incomplete success of fluoroscopy and CT-guided CA. This study involved 154 patients with 205 lesions, including 73 males and 81 females. Two studies [35,39] reported a mean age of 60.4 years. All patients were followed up for an average of 20.7 months. The most common primary cancer source was breast cancer (*n* = 41), followed by lung cancer (*n* = 33) and thyroid cancer (*n* = 21). The location of the spinal lesions was reported in this article (C = 58, T = 91, L = 48, S and coccyx = 12).

##### Demographic and Clinical Data of Fluoroscopy and CT-Guided RFA and CA (1)

One article reported less than a complete success rate for fluoroscopy and CT–guided RFA and CA [44] due to radiculopathy in one of the patients. It involved 21 patients with 36 lesions. A mean age of 61.8 (range 30–84) for 13 male and 9 female patients was reported in this study, with a mean follow-up of 12 months. Lung, renal, and breast cancers were the most common primary sources (*n* = 8, 5, and 3, respectively). The spinal lesions were in the thoracic and lumbar spinal regions (T = 15 and L = 21).

#### 3.5.2. Demographic and Clinical Data of X-Ray, CT, and MRI-Guided Study (1)

**Figure 8:**
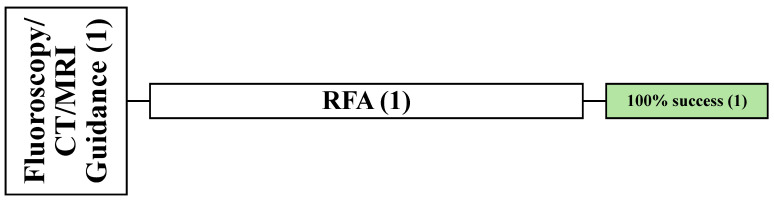
An overview of all patients undergoing X-ray-, CT-, and MRI-guided techniques. () the number of articles.

One retrospective article reported the complete success of X-ray, CT, and MRI–guided RFA in all 26 patients with 38 lesions [66]. Among these patients, 12 males and 14 females were involved with a mean age of 59.3 years (32–75 years) and were followed up for a range of 3–18 months. The most common primary cancers were breast, prostate, and lung cancer (6, 5, and 3 patients, respectively). The location of the spinal lesions was reported in this article as well (T = 10, L = 20, and S = 3) (Figure 8).

#### 3.5.3. Demographic and Clinical Data of Fluoroscopy and MRI-Guided Studies (1)

**Figure 9:**
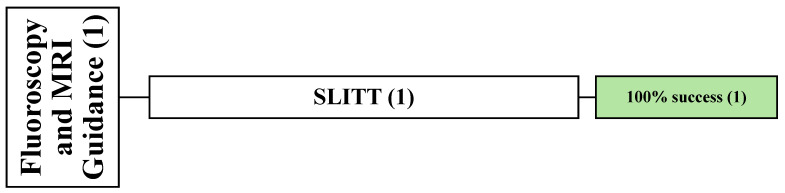
An overview of all patients undergoing fluoroscopy and MRI-guided techniques. () the number of articles.

One retrospective study reported the complete success of fluoroscopy and MRI-guided SLITT [58]. There were 13 patients, 8 males and 5 females, with a mean age of 60 years (40–81 years). All lesions were located at the thoracic spine and followed up for an average of 4.4 months (1.6–6.6 months). Thyroid and lung cancer were the most common primary cancer etiology reported (*n* = 4 each), followed by renal cancer (*n* = 2) (Figure 9).

#### 3.5.4. Demographic and Clinical Data of CT and MRI-Guided Studies (2)

**Figure 10:**
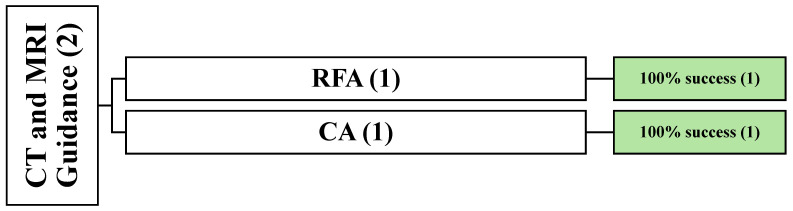
An overview of all patients undergoing CT and MRI-guided techniques. () the number of articles.

Two studies reported CT and MRI-guided techniques in 18 patients with 21 lesions [74,76]. One prospective article used CA [74], and the other prospective cohort used RFA [76] (Figure 10).

##### Demographic and Clinical Data of CT and MRI-Guided CA (1)

One prospective study reported a 100% success rate of CT and MRI–guided CA in 14 patients with 17 lesions [74]. Among these patients, six males and eight females were involved, with a mean age of 54.5 years (range: 35–81 years), and were followed up for an average of 9.8 months. The most common primary cancers were lung, breast, and renal (4, 3, and 3 patients, respectively). The location of the spinal lesions was reported in this article as well (cervical = 1, thoracic = 10, lumbar = 1, and sacral = 2).

##### Demographic and Clinical Data of CT and MRI-Guided RFA (1)

The last cohort reported complete thermal ablation (TA) success guided by CBCT-MRI fusion [76]. In total, four patients were involved and had lesions at the thoracic and lumbar levels (two each). They were all followed up for an average of 18 months. The primary cancer etiologies were Hodgkin lymphoma (*n* = 3) and prostate cancer (*n* = 1) (Figure 6).

## 4. Discussion

This systematic review aimed to evaluate the effectiveness of different imaging modalities—CT, fluoroscopy, MRI, and mixed techniques—in guiding thermal ablation procedures for treating spinal metastases. Overall, CT and fluoroscopy were the most employed modalities, while MRI guidance was less frequently used but offered distinct advantages in some instances.

As reported in a systematic review by Chen et al., the male-to-female gender distribution ratio was 0.86:1 [81]. Our study reported a similar ratio between male and female patients, with slightly more female patients affected (ratio: 0.96:1).

The mean age of patients undergoing thermal ablation of spinal metastasis was 60.5 years. In two studies reported by Tomasian et al. and Marini et al., the mean age of patients was 60 and 61.6 years, respectively, which is similar to our findings [24,82]. The advanced age of presentation is due to the comprehensive advancement in cancer therapies that extend life expectancy, providing an extended time interval for the primary tumors to metastasize and spread to the spinal column [83,84].

The most common primary cancer etiology reported in previous studies was lung and breast cancer, accounting for 21% and 19% of the total population, respectively [85]. Similarly, lung and breast cancer were the most common primary tumor etiologies, accounting for 24.4% and 20.8% of the cases involved, respectively.

CT guidance was utilized in 12 studies and was associated with high success rates across multiple ablation techniques, particularly in RFA and MWA. CT-guided RFA achieved complete success in 75% of studies, which aligns with its superior ability to provide precise anatomical visualization and accurate needle placement. CT-guided RFA, across four studies, demonstrated a consistent success rate, with an average follow-up of 12.6 months [48,68,71]. This high efficacy rate is likely due to CT’s excellent spatial resolution, which allows clinicians to target lesions while minimizing damage to adjacent critical structures effectively.

CT-guided MWA was similarly effective, with one study reporting a 100% success rate in 82 patients, but complications such as neural damage and skin burns were observed in other studies [38,61]. These complications highlight the need for careful procedural planning and monitoring during MWA, as the larger ablation zones produced by microwave energy may increase the risk of collateral damage. However, CT’s ability to visualize bone and soft tissues mitigates many of these risks when used appropriately.

CA guided by CT demonstrated mixed results, with two studies reporting incomplete success and postoperative complications such as radiculopathy in a few patients [50,57]. Despite CT’s effectiveness in guiding ablation, the inherent risk of nerve damage associated with cryoablation must be carefully managed with thermoprotective measures.

Fluoroscopy was used in 12 studies, primarily in conjunction with RFA and MWA. Fluoroscopy-guided RFA showed strong results, with a 100% success rate across 10 studies covering 300 patients with 399 lesions [21,47,49,53,54,62,63,64,69,79]. Its real-time imaging capability makes it particularly useful for continuous intraoperative monitoring and quick adjustments during the procedure. However, fluoroscopy provides limited soft-tissue contrast compared to CT, which may explain the slightly higher complication rates observed in some studies, resulting in complications such as nerve injury and incomplete tumor control [78,79].

Fluoroscopy-guided MWA also demonstrated efficacy, with one study reporting a 100% success rate, although complications such as nerve injury and recurrence were noted in others [31,78]. Given spinal metastases’ dynamic nature and proximity to critical structures, the absence of detailed soft-tissue contrast in fluoroscopy may pose challenges in guiding more complex ablations like MWA.

Only one study utilized MRI guidance, which reported complete success in 11 patients undergoing SLITT [55]. MRI’s ability to provide superior soft-tissue contrast and real-time visualization of ablation zones makes it valuable in more intricate cases where tumors are close to vital neural or vascular structures. Despite these advantages, the logistical complexity and limited availability of MRI-guided procedures have restricted its broader adoption in spinal metastasis treatment. However, the reduced complication rates and improved targeting accuracy observed in the MRI-guided study suggest that it may benefit certain patient groups significantly.

Twenty-six studies employed a combination of imaging modalities, including CT, MRI, and fluoroscopy. Mixed guidance was instrumental in complex cases, where each modality could complement the limitations of the others. For example, CT provided precise anatomical guidance, while MRI allowed for better visualization of soft tissue and real-time monitoring of ablation zones. The use of CBCT and MRI fusion technologies in some studies demonstrated improved accuracy in tumor targeting, particularly in difficult-to-reach areas of the spine [46,75]. This combination reduced the risk of thermal injury to adjacent structures, though more extensive studies are needed to confirm its broader applicability.

Across all imaging modalities, the overall complication rate was low, with most complications being self-limiting or managed conservatively. Thermal damage to adjacent tissues (e.g., nerve injury) and recurrence of tumor were the most frequently reported complications, particularly in fluoroscopy-guided procedures. CT-guided ablations had fewer complications due to the precise targeting of lesions. MRI-guided procedures showed the lowest complication rates, likely due to the superior soft-tissue contrast and real-time feedback provided during the procedure.

Although neural complications were relatively rare across the reviewed studies, they were observed in multiple ablation techniques, regardless of the imaging modality. This indicates that the risk of nerve injury persists, irrespective of whether CT, MRI, or fluoroscopy is employed to guide the procedure. Given the proximity of spinal metastases to critical neural structures, even minor inaccuracies in electrode placement or thermal monitoring can lead to significant complications. For this reason, we advocate for using CBCT-MRI fusion techniques, which combine the superior anatomical detail of CT with the soft-tissue contrast and real-time feedback provided by MRI. This hybrid approach allows for more precise electrode placement within the tumor while reducing the risk of overheating adjacent healthy tissues, especially neural components. By utilizing the strengths of both modalities, CBCT-MRI fusion may significantly decrease the incidence of neural complications and improve overall procedural safety.

However, this review has some limitations, notably the high heterogeneity among included studies regarding design, patient demographics, and follow-up durations, which complicates the direct comparison of findings. The predominance of retrospective studies and limited randomized controlled trials restrict the strength of conclusions, especially regarding the long-term efficacy and safety of imaging modalities for guiding thermal ablation. Additionally, a lack of extended follow-up data across studies limits insights into the durability of tumor control and potential delayed complications. While mixed imaging techniques, such as CBCT-MRI fusion, demonstrate potential, their clinical relevance remains to be validated in larger prospective trials. Future studies with rigorous methodologies are needed to address these gaps and optimize thermal ablation guidance for spinal metastases.

## 5. Conclusions

In conclusion, CT guidance remains the most widely used and effective imaging modality for guiding thermal ablation in spinal metastasis treatment, owing to its high spatial resolution and ability to visualize bone and soft tissue. While useful for real-time adjustments, fluoroscopy shows limitations in soft-tissue contrast, which can lead to higher complication rates in more complex procedures. Although less frequently used, MRI guidance offers superior soft-tissue visualization and may reduce complications in specific cases, especially in tumors near critical structures. Mixed imaging modalities, such as CBCT-MRI fusion, show promise in improving accuracy and minimizing risks, although further studies are needed to validate their efficacy in larger patient populations.

Future research should focus on directly comparing the outcomes of these imaging modalities in larger cohorts, with particular emphasis on long-term tumor control and complication rates. This will provide more precise guidance on when to select each imaging modality based on patient and tumor characteristics.

## Figures and Tables

**Table 1:** Detailed search strategy for PubMed, SCOPUS, and WOS.

	Equation	Records Identified (*n*)	Filters
PubMed	(Thermal ablation OR Ablation techniques OR Local thermal Treatment OR Minimally Invasive Surgical Procedures OR Percutaneous Thermal Ablation) AND (Spinal column Tumor OR Spinal column mass OR Spinal column Neoplasm OR Spinal column metastasis) AND (Ultrasound OR CBCT OR X-ray OR X-ray computed OR CT OR Computed Tomography OR Tomography OR MRI OR Magnetic Resonance Imaging OR Imaging modalities OR Imaging diagnostics OR Diagnostic Imaging OR Radiological)	1238	2000–2024 Language—English
OVID		2053	Language—English
Google Scholar		427	2000–2024 Articles
Web of Science		15	2000–2024 Articles

## Data Availability

Data sharing is not applicable to this article.
